# Rush Medical College—Annual Commencement

**Published:** 1858-03

**Authors:** 


### RUSH MEDICAL COLLEGE—ANNUAL COMMENCEMENT.

The fifteenth annual commencement in Rush Medical College was held in the College Hall, on the afternoon of the 17th of February. The hall was well filled at the hour appointed; and after the opening prayer by the Rev. Mr. Rice, several members of the graduating class were called upon to read their inaugural thesis.

The degree of Doctor of Medicine was then conferred on the following gentlemen, viz:

| | |
|---|---|
| Chas. J. Keegan, | S. B. Davis, |
| D. B. Montgomery, | J. T. Pearman, |
| Owen Wright, | J. B. Wilson, |
| O. B. Ormsby, | J. B. Earl, |
| Benj. F. Swofford, | W. B. Harl, |
| J. L. Potter, | Wm. H. Rockwell, |
| L. B. Brown, | Benj. Durham, |
| Freeman Clark, | J. S. Pashley, |
| L. Brookhart, | John O'Connors, |
| A. J. Miller, | J. Slack, |
| T. C. Jennings, | Thos. Winston, |
| B. F. Ross, | P. Corcoran, |
| B. F. Keith, | C. N. Ellinwood, |
| Eli York, | J. N. Green, |
| J. D. Gray, | Allen Heavenridge, |
| L. D. Smedley, | Wm. Somers, |
| J. R. Webster, | R. C. Black, |
| C. V. Snow, | Willis L. May. |

The honorary degree of Doctor of Medicine was conferred on Dr. Solomon Davis, of Columbus, Indiana, and Dr. Waldo W. Lake, of Milwaukee, Wisconsin.

The ceremony of conferring the degrees being concluded, a well written and very interesting valedictory address to the graduates was delivered by Prof. W. H. Byford. It was chiefly devoted to the elucidation of those ethical rules and principles which should govern the practitioner in all the relations of life. The positions taken were just; the illustrations happy; and the lessons inculcated impressive and salutary.

The ceremonies properly belonging to the public commencement having been concluded, the graduates and alumni of the college, together with the faculty and invited guests, retired to the museum room, where a collation had been prepared by Messrs. Anderson & Brother, and spent a couple of hours in the most agreeable and social manner. The free use of the bounties provided for the physical man, was interspersed with an equally free flow of wit, social hilarity, and intellectual enjoyment. At an early hour the company separated, feeling that the occasion would be remembered as a green spot in the diversified field of human life.

Thus closed another prosperous, and, we trust, profitable annual course of instruction in Rush Medical College. In looking over the names on the register, we find quite a number absent, who had intended to attend the course, but who were prevented by the financial embarrassment that swept like a pestilence over the country during the past autumn, and interposed a barrier in the way of the accomplishment of the plans and expectations of men in every relation of life. Still we had the average number of students and graduates, and their uniformly correct deportment and assiduous attention to instruction, both in the college and hospital, rendered the duties devolving on the faculty as pleasant as they were arduous.

